# Effect of Comorbidities on the Infection Rate and Severity of COVID-19: Nationwide Cohort Study With Propensity Score Matching

**DOI:** 10.2196/35025

**Published:** 2022-11-18

**Authors:** Jiyong Kim, Seong Hun Park, Jong Moon Kim

**Affiliations:** 1 Department of Rehabilitation Inje University Ilsanpaik Hospital Goyang Republic of Korea; 2 Statistical analysis company HYMS Gwangju Republic of Korea; 3 Department of Rehabilitation Medicine CHA Bundang Medical Center CHA University Seongnam Republic of Korea; 4 Department of Medical Informatics Big Data Center CHA Bundang Medical Center CHA University Seongnam Republic of Korea

**Keywords:** COVID-19, comorbidity, infection rate, severity of illness index, hyperlipidemia

## Abstract

**Background:**

A vaccine against COVID-19 has been developed; however, COVID-19 transmission continues. Although there have been many studies of comorbidities that have important roles in COVID-19, some studies have reported contradictory results.

**Objective:**

This study was conducted using real-world data from COVID-19 patients in South Korea and aimed to investigate the impact of patient demographics and comorbidities on the infection rate and severity of COVID-19.

**Methods:**

Data were derived from a nationwide South Korean COVID-19 cohort study with propensity score (PS) matching. We included infected individuals who were COVID-19–positive between January 1, 2020, and May 30, 2020, and PS-matched uninfected controls. PS matching was performed to balance the baseline characteristics of each comorbidity and to adjust for potential confounders, such as age, sex, Charlson Comorbidity Index, medication, and other comorbidities, that were matched with binary variables. The outcomes were the confirmed comorbidities affecting the infection rate and severity of COVID-19. The endpoints were COVID-19 positivity and severe clinical outcomes of COVID-19 (such as tracheostomy, continuous renal replacement therapy, intensive care unit admission, ventilator use, cardiopulmonary resuscitation, and death).

**Results:**

The COVID-19 cohort with PS matching included 8070 individuals with positive COVID-19 test results and 8070 matched controls. The proportions of patients in the severe group were higher for individuals 60 years or older (severe clinical outcomes for those 60 years or older, 16.52%; severe clinical outcomes for those of other ages, 2.12%), those insured with Medicaid (Medicaid, 10.81%; other insurance, 5.61%), and those with disabilities (with disabilities, 18.26%; without disabilities, 5.07%). The COVID-19 infection rate was high for patients with pulmonary disease (odds ratio [OR] 1.88; 95% CI 1.70-2.03), dementia (OR 1.75; 95% CI 1.40-2.20), gastrointestinal disease (OR 1.74; 95% CI 1.62-1.88), stroke (OR 1.67; 95% CI 1.23-2.27), hepatobiliary disease (OR 1.31; 95% CI 1.19-1.44), diabetes mellitus (OR 1.28; 95% CI 1.16-1.43), and cardiovascular disease (OR 1.20; 95% CI 1.07-1.35). In contrast, it was lower for individuals with hyperlipidemia (OR 0.73; 95% CI 0.67-0.80), autoimmune disease (OR 0.73; 95% CI 0.60-0.89), and cancer (OR 0.73; 95% CI 0.62-0.86). The severity of COVID-19 was high for individuals with kidney disease (OR 5.59; 95% CI 2.48-12.63), hypertension (OR 2.92; 95% CI 1.91-4.47), dementia (OR 2.92; 95% CI 1.91-4.47), cancer (OR 1.84; 95% CI 1.15-2.94), pulmonary disease (OR 1.72; 95% CI 1.35-2.19), cardiovascular disease (OR 1.54; 95% CI 1.17-2.04), diabetes mellitus (OR 1.43; 95% CI 1.09-1.87), and psychotic disorders (OR 1.29; 95% CI 1.01-6.52). However, it was low for those with hyperlipidemia (OR 0.78; 95% CI 0.60-1.00).

**Conclusions:**

Upon PS matching considering the use of statins, it was concluded that people with hyperlipidemia could have lower infection rates and disease severity of COVID-19.

## Introduction

The World Health Organization declared that COVID-19 was a pandemic in March 2020. By August 2022, approximately 600 million individuals had been infected, and more than 6 million had died. Since then, vaccines and therapeutic agents for COVID-19 have been developed. However, the current number of individuals with COVID-19 is still the same as that 1 year ago because it has not yet been eradicated [1]. COVID-19 can result in an asymptomatic presentation or flu-like symptoms. Some patients are admitted to the hospital for conservative treatment, and some require intensive care unit admission. Moreover, some patients may die as a result of COVID-19 [2,3]. As the number of individuals with COVID-19 increases, it is important to identify those who are vulnerable to severe COVID-19 to effectively manage health care resources accordingly and to improve the prognosis [4,5].

Since the COVID-19 outbreak, many studies of the demographic factors that predispose individuals to infection and of the identification of comorbidities of infected individuals have been performed. Most studies have reported similar overall results; however, some results of these studies are contradictory [6-9]. These differences in results may be attributable to the diversity of patients and medical systems in various countries worldwide. Most previous studies on comorbidities analyzed the baseline characteristics of people infected with COVID-19 without considering the bias caused by various factors that influence COVID-19. For instance, to determine whether hyperlipidemia affects the severity of COVID-19, it is necessary to control for statins, which are often used by individuals with hyperlipidemia. Although some studies suggested that statins might have a role in reducing the severity of COVID-19 [10,11], most studies did not confirm the use of statins; they only reported the effect of hyperlipidemia [12-16]. Hence, it is difficult to accurately determine the effect of hyperlipidemia on the severity of COVID-19. We investigated the effects of patient comorbidities on the infection rate and severity of COVID-19. Bias was reduced by propensity score (PS) matching for various variables that may affect COVID-19. We also analyzed the demographic characteristics of patients with COVID-19.

## Methods

### Study Design and Participants

We conducted a large-scale cohort study using a South Korean National Health Insurance claims database [17]. In South Korea, all citizens are registered in the Korean National Health Insurance Service (KNHIS) database. The KNHIS uses a nationwide, large-scale database system including information regarding the diagnostic codes from the International Classification of Diseases (ICD)-10, the names of the procedures performed, prescription drugs, hospital information, direct medical costs of inpatient and outpatient treatments, and medical insurance premiums. Because all Koreans are given unique identification numbers at birth that are used in the KNHIS, the health records of patients are not duplicated nor omitted [18,19]. For COVID-19 studies, KNHIS provides a COVID-19 cohort that includes people infected with COVID-19 and a control group that had never been infected. From January 1, 2020, to May 31, 2020, disease codes B342, B972, U071, U072, MT043, and 3/02 were used to identify patients with confirmed COVID-19. Data from the control group of individuals who were not previously diagnosed with COVID-19 were adjusted for sex, age, and region of residence. Moreover, the number of participants in the control group was 15 times the number of confirmed COVID-19 cases.

### Ethical Considerations

This study was approved by the relevant institutional review board and research ethics committee (ISPAIK 2020-06-048-001). The need for written consent was formally waived by the ethics committee. This study used the NHIS-2020-1-328 database provided by the KNHIS in 2020.

### Study Population

**Figure 1:**
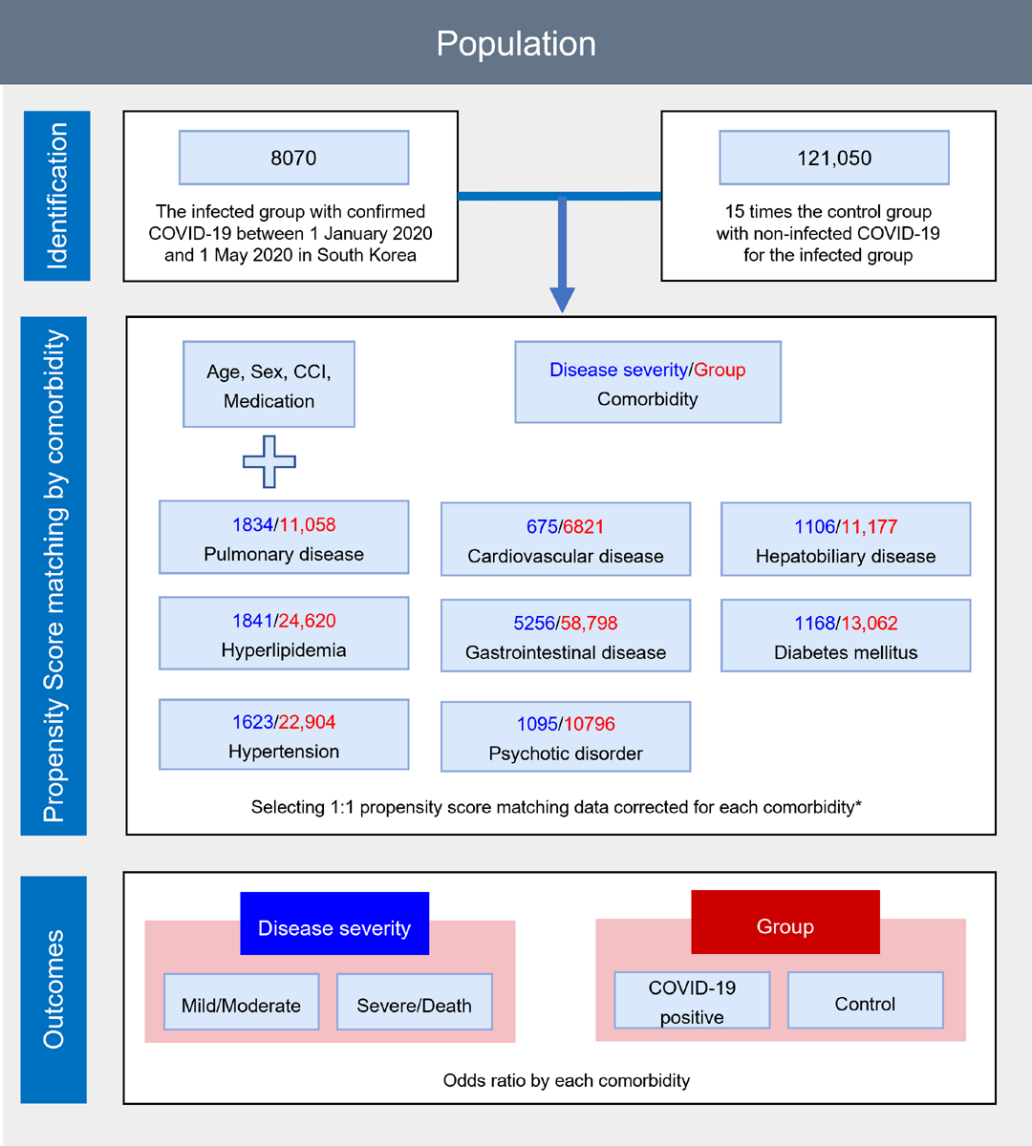
Disposition of patients in the KNHIS-COVID cohort (South Korea; January 1 to May 31, 2020). CCI: Charlson Comorbidity Index; KNHIS: Korean National Health Insurance Service.

In accordance with the World Health Organization guidelines, laboratory confirmation of COVID-19 was defined as a positive result of a real-time reverse-transcription polymerase chain reaction assay using a sample obtained with nasal and pharyngeal swabs [20]. We combined the claims-based data from the KNHIS between January 1, 2015, and May 31, 2020, and extracted information regarding age, sex, and region of residence from the insurance eligibility data (Figure 1). The Charlson Comorbidity Index (CCI) score was calculated using the ICD-10 codes and previously reported methods [21]. Certain underlying medications and diseases with a high risk of serious illness attributable to SARS-CoV-2, which causes COVID-19, were studied and reported by the Centers for Disease Control and Prevention (CDC) and previous meta-analysis studies [6-9,22]. In these studies, we selected factors to use for PS matching in the analysis (Tables S1 and S2 in Multimedia Appendix 1). Only those (pulmonary disease, cardiovascular disease, hepatobiliary disease, hyperlipidemia, gastrointestinal disease, diabetes mellitus, hypertension, and psychotic disorder) with more than 500 people with COVID-19 were selected because a small number of people with corresponding comorbidities might cause statistical bias (Multimedia Appendix 2). A history of underlying diseases (pulmonary disease, cardiovascular disease, kidney disease, hepatobiliary disease, hyperlipidemia, gastrointestinal disease, diabetes mellitus, hypertension, psychotic disorder, dementia, stroke, neurologic disorder, autoimmune disease, and cancer) was confirmed by the assignment of at least two claims within 1 year using the appropriate ICD-10 code.

We used various PS matching methods for factors affecting COVID-19: (1) matching for age, sex, and CCI; (2) additional matching for comorbidities; and (3) additional matching for medications. Finally, the results from (3) were used (Tables S3 and S4 in Multimedia Appendix 1). The financial revenue of the National Health Insurance of Korea consists of contributions from the insured and government subsidies, which can be used to analyze socioeconomic status. The contributions to the National Health Insurance differ according to the family income level. The higher the income, the greater the contribution to the National Health Insurance. Income was divided into 5 categories for the purpose of statistical analyses. The first category is Medicaid, and the successive categories include progressively higher (by 25%) income groups. Disability grades were categorized as mild or severe based on the KNHIS database information for people registered with the Korean government.

### Outcomes

To determine the severity of disease according to the demographic factors of COVID-19–infected patients, the severity scale was divided into the following 4 grades: mild, moderate, severe, and death. In South Korea, patients with asymptomatic or mild symptoms are discharged when a negative COVID-19 test result is confirmed 2 weeks after hospitalization. This time period also corresponds to the period of self-isolation. When we checked the hospitalization period of COVID-19–infected patients, the hospitalization period peaked on day 16 and decreased thereafter. Based on this result, a hospitalization period of ≤16 days was defined as the mild grade corresponding to asymptomatic or mild symptoms. The severe grade was defined as the need for tracheostomy, continuous renal replacement therapy, intensive care unit admission, ventilator use, and cardiopulmonary resuscitation. The moderate grade was defined as a hospitalization period >16 days but not requiring treatment corresponding to the severe grade.

The primary aim of this study was to compare the severity grades of the COVID-19–infected and control groups based on demographic factors, comorbidities, and complications. The secondary aim was to perform PS matching for comparisons. We identified the infection rate and severity (severe and death or mild and moderate) of COVID-19 according to the comorbid conditions.

### Statistical Analysis

We performed PS matching to balance the baseline characteristics of each comorbidity (existence or nonexistence) and to adjust for potential confounders. Because we focused on each comorbidity, PS matching was performed for each comorbidity. The PS was estimated using a logistic regression model and calculating the predicted probability of covariates. Age and CCI (0, 1, or ≥2) were matched with continuous variables. Sex, medication, and other comorbidities were matched with binary variables. We assessed the PS matching of the comorbidity existence using a 1:1 ratio, the greedy nearest neighbor algorithm, and a scale with a caliper of 0.25 (Multimedia Appendix 2). Data obtained after PS matching were analyzed by calculating the odds ratios (ORs) with 95% CIs for the infection rate and severity (severe and death or mild and moderate) of COVID-19. Statistical analyses were performed using SAS version 9.4 (SAS Institute Inc, Cary, NC).

### Patient and Public Involvement

No patient was directly involved in designing the study question or in conducting the study. No patients were asked for advice regarding the interpretation or writing of the results. There are no plans to involve patients or the relevant patient community in the dissemination of study findings at this time.

## Results

### Clinical Characteristics of the Study Population

A total of 8070 individuals had positive COVID-19 results according to the reverse-transcription polymerase chain reaction assay. We identified 121,050 uninfected individuals as control participants (Multimedia Appendix 2). The demographic characteristics of the entire cohort are displayed in Table 1. The COVID-19 severity grade was mild for 2419 (2419/8070, 29.98%) individuals, moderate for 5160 (5160/8070, 63.94%) individuals, severe for 254 (254/8070, 3.15%) individuals, and death for 237 (237/8070, 2.94%) individuals. Among the total sample of infected individuals, 3236 (3236/8070, 40.10%) were male. Most patients were in their fifth (1567/8070, 19.42%) or sixth (1199/8070, 14.86%) decade of life. In terms of the medical insurance grade, which indicates socioeconomic status, those receiving Medicaid had high rates of severe grade and death. However, there were no obvious trends for the other grades. Individuals with disabilities had more severe infections and a much higher case fatality rate (Table 1). Those with COVID-19 had a medical history of gastrointestinal disease (n=5256), pulmonary disease (n=2539), hyperlipidemia (n=1841), and hypertension (n=1623). The case fatality rate was high for individuals with dementia (74/235, 31.5%), kidney disease (25/86, 29%), and cardiovascular disease (110/675, 16.3%; Table 2). After COVID-19 was confirmed, gastrointestinal disease (n=2912), pulmonary disease (n=2398), and hepatobiliary disease (n=1248) were the most common complications (Table 3).

**Table 1:** Baseline characteristics of the study population, including those infected (n=8070) and uninfected (n=121,050; controls) with COVID-19 in the Korean National Health Insurance Service (KNHIS)-COVID cohort (South Korea; January 1, 2020, to May 31, 2020).

Variables		Severity of COVID-19, n (%)					Total COVID-19 cases, n (%)		Controls^a,b^, n (%)
		Mild	Moderate	Severe	Death				
**Sex**									
	Male	894 (27.63)	2073 (64.06)	135 (4.17)	134 (4.14)	3236 (40.10)		48,540 (40.10)	
	Female	1525 (31.55)	3087 (63.86)	119 (2.46)	103 (2.13)	4834 (59.90)		72,510 (59.90)	
**Age (years)**									
	0-9	32 (39.51)	45 (55.56)	4 (4.94)	0 (0.00)	81 (1.00)		1215 (1.00)	
	10-19	77 (27.90)	195 (70.65)	4 (1.45)	0 (0.00)	276 (3.42)		4140 (3.42)	
	20-29	697 (33.88)	1342 (65.24)	18 (0.88)	0 (0.00)	2057 (25.49)		30,855 (25.49)	
	30-39	273 (32.81)	541 (65.02)	17 (2.04)	1 (0.12)	832 (10.31)		12,480 (10.31)	
	40-49	358 (34.56)	655 (63.22)	20 (1.93)	3 (0.29)	1036 (12.84)		15,540 (12.84)	
	50-59	504 (32.16)	1006 (64.20)	43 (2.74)	14 (0.89)	1567 (19.42)		23,505 (19.42)	
	60-69	322 (26.86)	776 (64.72)	66 (5.50)	35 (2.92)	1199 (14.86)		17,985 (14.86)	
	70-79	122 (19.77)	389 (63.05)	40 (6.48)	66 (10.70)	617 (7.65)		9255 (7.65)	
	≥80	34 (8.40)	211 (52.10)	42 (10.37)	118 (29.14)	405 (5.02)		6075 (5.02)	
**Medical insurance^a^**									
	Medicaid	186 (27.56)	416 (61.63)	31 (4.59)	42 (6.22)	675 (8.36)		4424 (3.65)	
	Grade 1	604 (32.95)	1146 (62.52)	44 (2.40)	39 (2.13)	1833 (22.71)		26,258 (21.69)	
	Grade 2	462 (30.84)	971 (64.82)	36 (2.40)	29 (1.94)	1498 (18.56)		24,270 (20.05)	
	Grade 3	484 (29.02)	1081 (64.81)	58 (3.48)	45 (2.70)	1668 (20.67)		27,521 (22.74)	
	Grade 4	637 (28.07)	1475 (65.01)	79 (3.48)	78 (3.44)	2269 (28.12)		37,241 (30.76)	
**Disability grade**									
	Mild	65 (20.44)	192 (60.38)	28 (8.81)	33 (10.38)	318 (3.94)		4367 (3.61)	
	Severe	83 (27.57)	166 (55.15)	18 (5.98)	34 (11.30)	301 (3.73)		2275 (1.88)	
Total		2419 (29.98)	5160 (63.94)	254 (3.15)	237 (2.94)	8070 (100)		121,050 (100)	

^a^Participants from some specific groups, such as soldiers, were not included.

^b^The uninfected controls were adjusted for sex, age, and region, resulting in a figure equivalent to 15 times the number of confirmed COVID-19 cases in the KNHIS-COVID cohort.

**Table 2:** Baseline characteristics of comorbidities of the study population, including those infected with (n=8070) and not infected with (n=121,050; controls) COVID-19 in the Korean National Health Insurance Service (KNHIS)-COVID cohort (South Korea; January 1, 2020, to May 31, 2020).

Comorbidities	Severity of COVID-19, n (%)					Total COVID-19 cases, n (%)		Controls, n (%)
	Mild	Moderate	Severe	Death				
Pulmonary disease	501 (27.32)	1129 (61.56)	80 (4.36)	124 (6.76)	1834 (22.73)		11,058 (9.14)	
Cardiovascular disease	137 (20.44)	387 (57.33)	40 (5.93)	110 (16.30)	675 (8.36)		6821 (5.63)	
Kidney disease	15 (17.44)	37 (43.02)	9 (10.47)	25 (29.07)	86 (1.07)		1018 (0.84)	
Hepatobiliary disease	281 (25.41)	690 (62.39)	51 (4.62)	84 (7.59)	1106 (13.71)		10,177 (8.41)	
Hyperlipidemia	466 (25.31)	1158 (62.90)	97 (5.27)	120 (6.52)	1841 (22.81)		24,620 (20.34)	
Gastrointestinal disease	1559 (29.66)	3334 (63.43)	181 (3.44)	182 (3.46)	5256 (65.13)		58,798 (48.57)	
Diabetes mellitus	246 (12.50)	720 (61.64)	69 (5.91)	133 (11.39)	1168 (14.47)		13,062 (10.79)	
Hypertension	348 (21.44)	1010 (62.23)	95 (5.85)	170 (10.47)	1623 (20.11)		22,904 (18.92)	
Psychotic disorder	256 (23.38)	672 (61.37)	67 (6.12)	100 (9.13)	1095 (13.57)		10,796 (8.92)	
Dementia	19 (8.09)	116 (49.36)	26 (11.06)	74 (31.49)	235 (2.91)		1429 (1.18)	
Stroke	18 (15.13)	73 (61.34)	10 (8.40)	18 (15.13)	119 (1.47)		809 (0.67)	
Neurogenic disorder	25 (21.01)	75 (63.03)	4 (3.36)	15 (12.61)	119 (1.47)		1037 (0.86)	
Autoimmune disease	51 (28.33)	115 (63.89)	5 (2.78)	9 (5.00)	180 (2.23)		2423 (2.00)	
Cancer	55 (20.45)	158 (58.74)	19 (7.06)	37 (13.75)	269 (3.33)		3275 (2.71)	

**Table 3:** Baseline characteristics of complications of the study population, including those infected with (n=8070) and not infected with (n=121,050; controls) COVID-19 in the Korean National Health Insurance Service (KNHIS)-COVID cohort (South Korea; January 1, 2020, to May 31, 2020).

Complications	Severity of COVID-19, n (%)					Total COVID-19 cases, n (%)		Controls, n (%)
	Mild	Moderate	Severe	Death				
Pulmonary disease	542 (22.60)	1580 (65.89)	180 (7.51)	96 (4.00)	2398 (29.71)		2027 (1.67)	
Cardiovascular disease	161 (20.77)	475 (61.29)	85 (10.97)	54 (6.97)	775 (9.60)		1223 (1.01)	
Kidney disease	12 (11.01)	50 (45.87)	22 (20.18)	25 (22.94)	109 (1.35)		226 (0.19)	
Hepatobiliary disease	321 (25.72)	789 (63.22)	107 (8.57)	31 (2.48)	1248 (15.46)		4359 (3.60)	
Gastrointestinal disease	824 (28.30)	1906 (65.45)	129 (4.43)	53 (1.82)	2912 (36.08)		20,477 (16.92)	
Stroke	6 (13.33)	27 (60.00)	10 (22.22)	2 (4.44)	45 (0.56)		298 (0.25)	
Neurogenic disorder	21 (28.77)	40 (54.79)	12 (16.44)	3 (4.11)	73 (0.90)		169 (0.14)	
Sepsis	16 (10.46)	71 (46.41)	38 (24.84)	28 (18.30)	153 (1.90)		22 (0.02)	

### Risks of COVID-19 Positivity and Disease Severity According to Comorbidities

To identify differences according to comorbidity, predispositions were matched between the COVID-19–infected group and uninfected control group. No significant imbalances in the demographics and clinical characteristics were observed when they were assessed using the standardized mean difference within groups of PS-matched cohorts, which included the standardized mean difference of binary type variables <0.1. PS-matched ORs were checked for age, sex, CCI, medication, and comorbidities. When the control group and COVID-19–infected group were compared, COVID-19 was likely to occur in individuals with a history of the diseases and medical conditions but not for those with a history of hyperlipidemia (OR 0.73; 95% CI 0.67-0.80), autoimmune disease (OR 0.73; 95% CI 0.60-0.89), or cancer (OR 0.73; 95% CI 0.62-0.86; Table 4). The severity grade was high for COVID-19–infected individuals with pulmonary disease (OR 1.72; 95% CI 1.35-2.19), cardiovascular disease (OR 1.54; 95% CI 1.17-2.04), kidney disease (OR 5.59; 95% CI 2.48-12.63), diabetes mellitus (OR 1.43; 95% CI 1.09-1.87), hypertension (OR 1.63; 95% CI 1.23-2.15), psychotic disorder (OR 1.29; 95% CI 1.01-6.52), dementia (OR 2.92; 95% CI 1.91-4.47), or cancer (OR 1.84; 95% CI 1.15-2.94). However, the severity grade was low for COVID-19–infected individuals with hyperlipidemia (OR 0.70; 95% CI 0.55-0.90; Table 5 and Table S5 in Multimedia Appendix 1).

**Table 4:** Propensity score–matched (age, sex, Charlson Comorbidity Index, medications, and comorbidities) baseline characteristics and COVID-19 infection positivity rates according to comorbidity in the Korean National Health Insurance Service (KNHIS)-COVID cohort (South Korea; January 1, 2020, to May 31, 2020).

Comorbidities	Group^a^, n		Odds ratio (95% CI)
	COVID-19	Control	
Pulmonary disease^b^	2880	22,900	1.88 (1.70-2.03)
Cardiovascular disease^b^	1245	13,733	1.20 (1.07-1.35)
Kidney disease	171	2037	1.01 (0.74-1.39)
Hepatobiliary disease^b^	1959	20,505	1.31 (1.19-1.44)
Hyperlipidemia^b^	2375	25,663	0.73 (0.67-0.80)
Gastrointestinal disease^b^	3164	43,370	1.74 (1.62-1.88)
Diabetes mellitus^b^	1544	16,468	1.28 (1.16-1.43)
Hypertension	1483	16,473	1.04 (0.93-1.15)
Psychotic disorder	2122	21,458	1.06 (0.97-1.16)
Dementia^b^	365	2753	1.75 (1.40-2.20)
Stroke^b^	194	1662	1.67 (1.23-2.27)
Neurologic disease	223	2089	1.16 (0.88-1.53)
Autoimmune disease^c^	421	4785	0.73 (0.60-0.89)
Cancer^c^	629	6459	0.73 (0.62-0.86)

^a^We assessed each propensity score–matched comorbidity using a 1:1 ratio for those in the COVID-19 and control groups.

^b^Comorbidity with more susceptibility to COVID-19.

^c^Comorbidity with less susceptibility to COVID-19.

**Table 5:** Propensity score–matched (age, sex, Charlson Comorbidity Index, medications, and comorbidities) baseline characteristics and clinical outcomes of COVID-19 among patients in the mild or moderate group and those in the severe or death group according to the comorbidity of patients with laboratory-confirmed COVID-19 infection in the Korean National Health Insurance Service (KNHIS)-COVID cohort (South Korea; January 1, 2020, to May 31, 2020).

Comorbidities	Severity^a^, n			Odds ratio (95% CI)
	Mild + moderate	Severe + death		
Pulmonary disease^b^	3031	307	1.72 (1.35-2.19)	
Cardiovascular disease^b^	1078	252	1.54 (1.17-2.04)	
Kidney disease^b^	129	43	5.59 (2.48-12.63)	
Hepatobiliary disease	1899	253	1.01 (0.78-1.31)	
Hyperlipidemia^c^	2204	270	0.78 (0.60-1.00)	
Gastrointestinal disease	3060	206	1.00 (0.75-1.33)	
Diabetes mellitus^b^	1465	259	1.43 (1.09-1.87)	
Hypertension^b^	1262	248	2.92 (1.91-4.47)	
Psychotic disorder^b^	1846	288	1.29 (1.01-6.52)	
Dementia^b^	305	137	2.92 (1.91-4.47)	
Stroke	188	50	1.36 (0.72-2.54)	
Neurologic disease	198	40	0.88 (0.45-1.75)	
Autoimmune disease	338	20	2.25 (0.92-6.52)	
Cancer^b^	448	88	1.84 (1.15-2.94)	

^a^We assessed each propensity score–matched comorbidity using a 1:1 ratio for those in the mild and moderate group and those in the severe and death group.

^b^Comorbidity with increasing COVID-19 severity.

^c^Comorbidity with decreasing COVID-19 severity.

## Discussion

### Principal Findings

This study was a retrospective cohort study conducted in South Korea from January 2020 to May 2020. It involved confirmed COVID-19 patients with medical insurance. Previous studies of the demographic factors of individuals with COVID-19 showed that male sex, old age, and low income were factors likely associated with COVID-19 with a high severity grade [23,24]. In this study, more women had COVID-19, but the severity grade of COVID-19 was higher for men; this was directly proportional to age, especially for men older than 70 years. All medical expenses for COVID-19 are paid for by the South Korea government; therefore, all patients, including those receiving Medicaid, received the same level of care for COVID-19. Although there was no difference in medical care, those with Medicaid had the lowest income level and a higher severity grade; however, there were no differences between the groups with grades 1 to 4 medical insurance. For individuals with disabilities, the incidence was slightly higher than that of the control group. However, the severity grade was much higher than that of other individuals infected with COVID-19.

Other studies of COVID-19 reported that SARS-CoV-2 binds to the angiotensin-converting enzyme 2 (ACE2) receptor through the viral structural spike protein at the onset of infection [25]. ACE2 is expressed to varying degrees in almost all human organs. ACE2 is highly expressed in cardiomyocytes, proximal tubule cells of the kidney, and bladder urinary tract cells. Additionally, it is abundantly expressed in intestinal cells of the small intestine, especially in the ileum [25-28]. Therefore, most critically ill patients with COVID-19 experience multiple organ injuries, including acute lung injuries, acute kidney injuries, cardiac injuries, hepatobiliary disease, and pneumothorax [29]. Therefore, to analyze the effect of each comorbidity on the COVID-19 infection severity grade, it is necessary to consider other comorbidities.

Each demographic factor, comorbidity, and medication may influence each other, resulting in different outcomes in terms of the infection rate and severity of COVID-19. When analyzing comorbidities with hypertension, the effect of hypertension on the infection rate and severity of COVID-19 experienced by an 80-year-old woman with asthma and that of a 30-year-old man without an underlying medical condition may be different. Accurate results can be obtained for sufficiently studied diseases by controlling for only important factors. However, in the case of understudied diseases, such as COVID-19, various factors should be considered. In this study, PS matching was performed for various factors that could affect COVID-19, to minimize bias. When selecting a factor for PS matching, in order to select objective data, data provided by the CDC and meta-analysis studies were used. However, there was a limit, as data may change as research on COVID-19 progresses. Most of the results obtained were similar to those of previously published studies; however, some results were conflicting. For people with cancer and autoimmune disease, infection rates were even lower; these results were possibly affected by reducing social contact because of the risk of COVID-19 infection. Exposure to COVID-19 is an important factor that can affect the infection rate of COVID-19. Individuals with hyperlipidemia had a low COVID-19 infection rate and low severity grade. Previous studies reported that hyperlipidemia should be managed to prevent COVID-19 because high cholesterol levels induce inflammation and increase ACE2 availability [30-32]. Moreover, the use of statins for patients with COVID-19 reduced mortality by interfering with the mevalonate pathway and because of their antiviral effects [10,11,33]. However, some studies have shown that people with low lipid levels are more susceptible to and have more severe COVID-19 infection [34-41]. A meta-analysis published in 2022 indicated that patients with severe COVID-19 had lower total cholesterol levels (pooled mean difference –10.4; 95% CI –18.7 to –2.2), low-density lipoprotein cholesterol levels (pooled mean difference –4.4; 95% CI –8.4 to –0.42), and high-density lipoprotein cholesterol levels (pooled mean difference –4.4; 95% CI –6.9 to –1.8) on admission compared with patients with non-severe disease [42]. This may be similar to the “obesity paradox,” which states that mild obesity is advantageous to improvements after stroke [43,44]. Mild obesity can withstand the systemic catabolic imbalance with impaired metabolic efficiency and body tissue degradation that occur after stroke. Hyperlipidemia may also have a role in minimizing the severity of COVID-19.

### Limitations

Our study has several limitations. As a limitation of most medical data studies, there is bias caused by confounding factors that may affect our results. When selecting factors for PS matching, information from the CDC and meta-analysis studies were used to select objective data, but these data may change as research on COVID-19 progresses. Further, we defined diseases based on the ICD codes provided in the insurance claims data. There may have been additional unmeasured confounders influencing our results, including genetic polymorphisms, smoking, body mass index, and exposure to the virus. In this study, the infection rate of COVID-19 may have been influenced by the degree of exposure to COVID-19, which may be an important factor in addition to the comorbidity factors. However, the influence of COVID-19 itself could be confirmed because the bias was less than that of previous studies. One race in South Korea comprises more than 95% of the population; hence, there was minimal racial bias compared with previous studies. Because the government funds the treatment for COVID-19 in South Korea and because the medical facilities for COVID-19 treatment are ubiquitous, there was minimal economic bias. The PS matching was performed for sex, age, CCI, comorbidity, and medication, including statins (standardized mean difference <0.1). Hence, selection bias was minimized. Therefore, more accurate information regarding the incidence of COVID-19 and its severity according to comorbidities was provided.

This study was based on data from patients who experienced COVID-19 during the early outbreak period; therefore, that strain may differ from the current strain of COVID-19. However, an accurate analysis of recent COVID-19 strains, including Omicron, is difficult because the effects of acquired or natural immunity and vaccination are mixed. Data at the time of its early onset can provide fundamental information, including regarding mutations that may occur in the future.

### Conclusions

Although the severity of COVID-19 has decreased, its hospitalization rate has not decreased significantly, and its burden on medical facilities continues; therefore, an analysis of comorbidities is still important. Therefore, many studies of comorbidities that affect COVID-19 have been published; however, some have reported conflicting results. This may be because various factors such as medication and comorbidities, in addition to demographic factors such as age and sex, affect the infection rate and severity of COVID-19. It is necessary to analyze as many factors as possible to obtain more accurate data regarding COVID-19. Based on the results of previous studies, this study tried to derive objective results by considering various factors affecting COVID-19. In conclusion, certain comorbidities known as risk factors in previous studies increase the infection rate and severity of COVID-19. However, hyperlipidemia decreases the infection rate and severity. These results can be utilized to effectively manage COVID-19.

## Appendix

Multimedia Appendix 1 Supplementary tables.

Multimedia Appendix 2 Statistical analysis method for the baseline characteristics of comorbidity and propensity score–matched infection rate and severity of COVID-19 in the Korean National Health Insurance Service (KNHIS)-COIVD cohort (South Korea; January 1, 2020, to May 31, 2020; pulmonary disease used as the example).

## Abbreviations

ACE2: angiotensin-converting enzyme 2
CCI: Charlson Comorbidity Index
CDC: Centers for Disease Control and Prevention
ICD: International Classification of Diseases
KNHIS: Korean National Health Insurance Service
NRF: National Research Foundation
OR: odds ratio
PS: propensity score
