# Supplementary figures and images for: Effect of Comorbidities on the Infection Rate and Severity of COVID-19: Nationwide Cohort Study With Propensity Score Matching
Source: JMIR Public Health Surveill. 2022 Nov 18;8(11):e35025. doi: 10.2196/35025 (PMC9678330; doi:10.2196/35025)

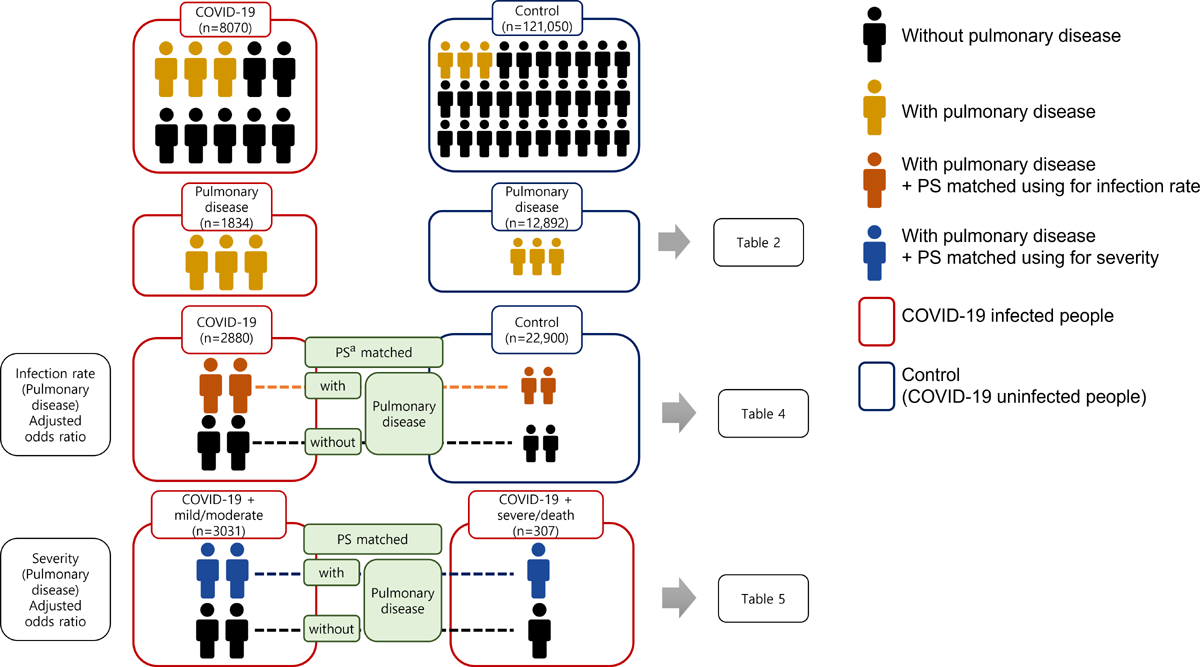

Supplement: Multimedia Appendix 2 [file publichealth_v8i11e35025_app2.png]
